# Vulvar Nonclear Cell Syringoma Associated with Pruritus and Diabetes Mellitus

**DOI:** 10.1155/2013/418794

**Published:** 2013-08-28

**Authors:** Gulsen Akoglu, Ibrahim Ibiloglu, Nezih Durmazlar

**Affiliations:** ^1^Dermatology Clinic, Ankara Cubuk Halil Sivgin State Hospital, 06760 Ankara, Turkey; ^2^Department of Pathology, Ankara Cubuk Halil Sivgin State Hospital, 06760 Ankara, Turkey; ^3^Department of Gynecology and Obstetrics, Ankara Cubuk Halil Sivgin State Hospital, 06760 Ankara, Turkey

## Abstract

*Background*. Syringoma is a benign eccrine sweat gland tumor, characterized by multiple skin colored or slightly yellowish papules. Vulvar syringoma without extragenital involvement is an extremely rare variant of syringoma. *Mail Observations*. Herein, we report a 40-year-old diabetic female patient who presented with only lichenified plaques on the vulvar region without any extragenital lesions. Diagnosis of vulvar syringoma was made depending on the characteristic double lined cystic enlargements and comma-like tails found in histopathological examination. Immunohistochemical stains for estrogen and progesterone receptors were negative. *Conclusion*. Vulvar syringoma may present with no obvious papules on lichenified plaques.

## 1. Introduction

Syringoma is a benign eccrine sweat gland tumor, usually presented with multiple skin colored papules on face, neck, and trunk with a female predominance. Syringomas usually involve face, neck, and trunk with a female predominance [1, 2]. However, vulvar syringoma without extragenital involvement is an extremely rare variant of syringoma [1]. Clinical suspicion and histopathological examination provide the true diagnosis of vulvar syringoma. Herein, we describe a 40-year-old diabetic female patient diagnosed as having vulvar syringoma with no obvious papules on lichenified plaques.

## 2. Case Report

A 40-year-old female patient presented with genital pruritus lasting for about 1 year. Pruritus was intense during the menstruation period. She denied oral contraceptive intake, seasonal change, or any contact hypersensitivity. There was no family history of similar complaints or any genital lesions. In the medical history, the patient had only peptic ulcer for about 2 years.

**Figure 1:**
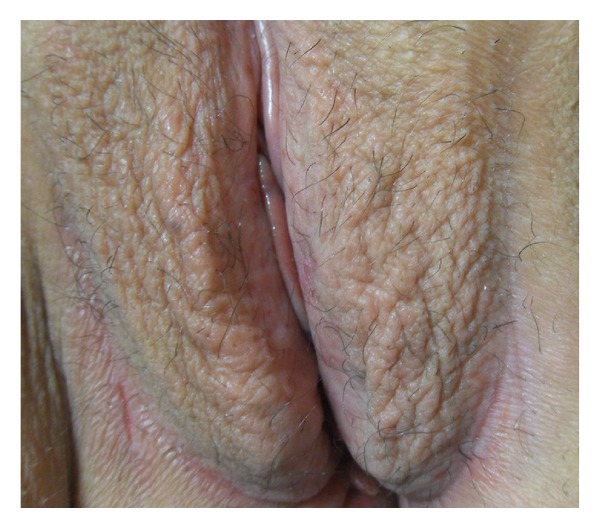
Lichenification on the vulvar region.

**Figure 2:**
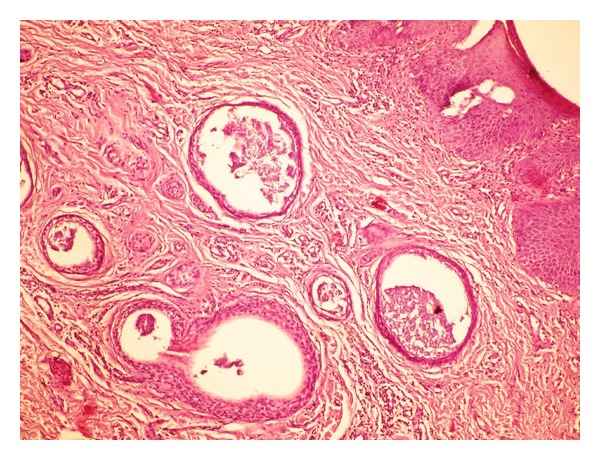
Tumoral lesion consisted of cystic enlargement of two-layered tadpole-like ductal epithelial structures including eosinophilic material, embedded in fibrous stroma (H&E ×200).

Dermatological examination revealed symmetrical lichenified plaques on bilateral labia majora and abrasion and erythema on the edges of the vulvar region (Figure 1). Otherwise body skin was normal, and she had no similar extragenital lesions. Papanicolaou smear of cervix and swabs from the vulvar region did not show any pathological findings and bacterial or fungal elements. Histopathological examination revealed multiple cystic enlargements, lined by two layers of cuboidal epithelium, some of which appeared as tadpole-like ductal epithelial structures including eosinophilic material, embedded in fibrotic stroma (Figure 2). Immunohistochemical stains for receptors of estrogen (ER) and progesterone (PR) were negative. Depending on these clinical and histopathological findings, the patient was diagnosed as having vulvar syringoma. Laboratory tests showed only high serum fasting glucose (280 mg/dL) and HbA1c levels (12.24 mmol/mL). It was the first time that she was diagnosed as type 2 diabetes mellitus (DM). Betamethasone dipropionate ointment and antihistamines were administered; however, pruritus partially regressed. The patient then was lost to followups. 

## 3. Discussion

Diagnosis of vulvar syringoma may be overlooked when papules are asymptomatic, and no other lesions are present in other sites of the body. On the contrary, large variants may be observed causing a serious cosmetic concern for the patients [3]. The present case demonstrated a distinct form of vulvar syringoma without extragenital involvement since typical syringoma papules were not visible on the lichenified plaques. The diagnosis of vulvar syringoma could be made only by histopathological examination. 

Vulvar syringoma should be in the differential diagnosis of pruritus vulva and vulvar papular lesions, such as Fox-Fordyce disease, epidermal cysts, milias, senile angiomas, condyloma acuminata, steatocystoma multiplex, vulvar idiopathic calcinosis, lymphangioma circumscriptum, and lichen simplex chronicus [1, 3]. Patients with vulvar syringoma may complain of severe itching [4–6]. Increased pruritus during menstruation and detection of ER and PR in some patients suggested that hormonal factors play role in the development of vulvar syringomas [4, 7, 8]. Pruritus was frequent in the largest series reported by Huang et al.; however, investigators did not detect positivity for ER and PR [5]. Nevertheless, failure to demonstrate these receptors still does not necessarily rule out the hormonal cause in our case, who is in child bearing age.

Treatment of vulvar syringomas with pruritus is challenging. Antihistamines and topical steroids may be ineffective in some cases. Electrodessication, excision, laser, or cryotherapy may provide favorable cosmetic results and regression of pruritus [5, 6].

Syringomas associated with endocrinopathy are limited in number. The eruptive and clear cell variants were mostly reported to be associated with DM [8, 9] The hormonal control of syringomas was suggested in the etiopathogenesis of especially the clear cell variants with DM [8, 10]. To the best of our knowledge, the presentation of nonclear cell variant of localized syringoma on the vulvar region associated with DM has not been reported before. The endocrinological abnormalities underlying DM might have predisposed the development of vulvar syringomas and probably contributed to more itching and changing in morphology.

## References

[1] Young AW, Herman EW, Tovell HMM (1980). Syringoma of the vulva: incidence, diagnosis, and cause of pruritus. *Obstetrics and Gynecology*.

[2] Jamalipour M, Heidarpour M, Rajabi P (2009). Generalized eruptive syringomas. *Indian Journal of Dermatology*.

[3] Dereli T, Turk BG, Kazandi AC (2007). Syringomas of the vulva. *International Journal of Gynecology and Obstetrics*.

[4] Yorganci A, Kale A, Dunder I, Ensari A, Sertcelik A (2000). Vulvar syringoma showing progesterone receptor positivity. *British Journal of Obstetrics and Gynaecology*.

[5] Huang Y, Chuang Y, Kuo T, Yang L, Hong H (2003). Vulvar syringoma: a clinicopathologic and immunohistologic study of 18 patients and results of treatment. *Journal of the American Academy of Dermatology*.

[6] Kavala M, Can B, Zindanci I (2008). Vulvar pruritus caused by syringoma of the vulva. *International Journal of Dermatology*.

[7] Wallace ML, Smoller BR (1995). Progesterone receptor positivity supports hormonal control of syringomas. *Journal of Cutaneous Pathology*.

[8] Timpanidis PC, Lakhani SR, Groves RW (2003). Progesterone receptor-positive eruptive syringoma associated with diabetes. *Journal of the American Academy of Dermatology*.

[9] Yoshimi N, Kurokawa I, Kakuno A, Tsubura A, Yamanishi K (2012). Case of generalized eruptive clear cell syringoma with diabetes mellitus. *Journal of Dermatology*.

[10] Shimizu A, Nagai Y, Ishikawa O (2000). Guess what! Clear cell syringoma. *European Journal of Dermatology*.

